# Dietary Omega-3 Fatty Acid Intake, Anxiety, and Depression Among University Students in the UK: An Exploratory Cross-Sectional Study

**DOI:** 10.3390/nu18172858

**Published:** 2026-09-01

**Authors:** Mine Mumcu, John K. Lodge, Hasan Kaan Kavsara

**Affiliations:** 1 School of Human Sciences, London Metropolitan University, London N7 8DB, UK; minemumcu16@hotmail.com; 2 Department of Nutrition and Dietetics, Faculty of Health Sciences, Yeditepe University, Istanbul 34755, Türkiye; kaan.kavsara@yeditepe.edu.tr

**Keywords:** Omega-3 fatty acids, EPA, DHA, ALA, anxiety, depression, university students, dietary intake

## Abstract

Background: Omega-3 fatty acids may be relevant to mental health, but evidence among university students remains limited. This small exploratory cross-sectional study examined associations between estimated dietary omega-3 intake and anxiety and depressive symptoms among university students in the UK. Methods: Data were collected between February and May 2025. Habitual dietary EPA + DHA and alpha-linolenic acid (ALA) intakes were estimated using an adapted omega-3 food-frequency questionnaire, while 24-h dietary recall was used to estimate energy intake. Anxiety and depressive symptoms were assessed using the GAD-7 and PHQ-9. The primary association between estimated dietary EPA + DHA intake and GAD-7 score was examined using linear regression adjusted for age, sex, energy intake, and alcohol use. Analyses involving ALA and PHQ-9 were exploratory. Results: Of 96 students recruited, 50 (52.1%) were analysed after 46 exclusions, most commonly for omega-3 supplement use (*n* = 21) or diagnosed mental-health conditions (*n* = 16). Mean GAD-7 and PHQ-9 scores were 6.66 ± 4.73 and 7.02 ± 4.76, respectively. In the fully adjusted model, each additional 100 mg/day of estimated dietary EPA + DHA intake was associated with a 0.325-point lower GAD-7 score, but the association was not statistically significant (B = −0.325; 95% CI: −0.725 to 0.074; *p* = 0.108). Exploratory analyses found no significant associations of estimated dietary EPA + DHA or ALA intake with anxiety or depressive symptoms. The inverse coefficient lost statistical significance after full adjustment, and the log-transformed coefficient reversed direction and remained non-significant, arguing against a consistent dose–response relationship. Conclusions: Estimated dietary EPA + DHA intake showed an inverse but inconclusive association with anxiety symptoms in this small exploratory sample. These preliminary findings are hypothesis-generating and require confirmation in larger longitudinal studies using repeated dietary assessments and biomarkers of omega-3 status.

## 1. Introduction

Anxiety and depression are common mental health concerns among university students and may adversely affect well-being, academic performance, social functioning, and overall quality of life [1]. University students may be particularly vulnerable to psychological distress because of academic workload, financial concerns, social transitions, and uncertainty about future employment [2]. Although psychological and pharmacological interventions remain central to the management of anxiety and depression, increasing attention has been directed towards potentially modifiable lifestyle factors, including diet quality and specific nutrient intakes [3].

Within nutritional psychiatry, omega-3 polyunsaturated fatty acids have received considerable interest in relation to depression and other mental-health outcomes [4,5,6]. The principal omega-3 fatty acids relevant to human health are eicosapentaenoic acid (EPA), docosahexaenoic acid (DHA), and alpha-linolenic acid (ALA). EPA and DHA, obtained primarily from oily fish and seafood, have important roles in neuronal membrane structure and in the generation of lipid mediators involved in inflammatory regulation [7]. Randomised-trial evidence has also evaluated long-chain omega-3 fatty acids in relation to anxiety and depression [8]. Proposed mechanisms relevant to these outcomes include effects on neurotransmission, inflammatory signalling, oxidative stress, and neuroplasticity [9]. A separate dose–response meta-analysis has specifically examined omega-3 supplementation in relation to anxiety symptoms [10].

ALA is an essential plant-derived omega-3 fatty acid found in foods such as walnuts, flaxseeds, chia seeds, hemp seeds, and certain vegetable oils [11]. Although ALA can be converted into EPA and DHA, this conversion is limited in humans; direct dietary intake of EPA and DHA therefore contributes importantly to long-chain omega-3 status [11]. Questionnaire-based screening instruments have also been developed to estimate dietary omega-3 fatty acid intake [12]. International dietary guidance generally encourages regular consumption of oily fish or EPA and DHA for general health [13,14]. These recommendations represent general-health reference values rather than thresholds for preventing anxiety or depression. Nevertheless, consumption of omega-3-rich foods may be suboptimal among young adults and university students [15].

Previous observational and intervention studies have examined the relationship between omega-3 fatty acids and anxiety and depressive symptoms, but their findings remain inconsistent [16,17]. Some studies have reported inverse associations between DHA or total omega-3 intake and anxiety symptoms [18], while meta-analytic evidence has suggested a possible inverse association between fish consumption and depression risk [19]. However, much of the available evidence is derived from supplementation studies, clinical populations, or assessments of broader dietary patterns. Evidence concerning habitual dietary intake of specific omega-3 fatty acids among non-clinical university students remains limited. Moreover, EPA + DHA and ALA have frequently not been examined separately despite important differences in their dietary sources, metabolism, and biological activity.

Therefore, the primary objective of this small exploratory cross-sectional study was to examine the association between estimated habitual dietary EPA + DHA intake and anxiety symptom severity, assessed using the GAD-7, among university students in the UK. Given the limited sample size and exploratory design, these analyses were intended to provide preliminary, hypothesis-generating evidence rather than confirmatory inference. The secondary exploratory objective was to examine associations involving estimated ALA intake and depressive symptom severity assessed using the PHQ-9, as well as differences according to reference-based omega-3 intake categories. It was hypothesised that higher estimated EPA + DHA intake would be associated with lower GAD-7 scores. Analyses involving estimated dietary ALA intake and PHQ-9 scores were considered exploratory.

## 2. Materials and Methods

### 2.1. Study Design

This exploratory cross-sectional study was conducted between February and May 2025 to examine the association between dietary omega-3 fatty acid intake and self-reported symptoms of anxiety and depression among university students in the UK. Data were collected using an online questionnaire that included a food frequency questionnaire, a 24-h dietary recall, validated mental health screening tools, and demographic and lifestyle questions.

### 2.2. Participants and Recruitment

Participants were recruited using convenience and snowball sampling. The questionnaire link was distributed through social media platforms commonly used by university students in the UK, including Instagram, LinkedIn, and WhatsApp. In-person recruitment was also undertaken using QR codes displayed in frequently visited university locations, such as libraries and cafeterias. Because this non-probability recruitment strategy did not use a population-based sampling frame, the resulting sample should be regarded as a convenience sample and not as representative of the wider UK university-student population.

Eligible participants were students aged 18 years or older who were currently enrolled at a UK university, resided in the UK, and were able to provide informed consent. Eligibility was assessed using self-reported questionnaire responses. Participants reporting gallbladder disease, pancreatitis, inflammatory bowel disease, or coeliac disease were excluded because these conditions may affect fat digestion or nutrient absorption. Irritable bowel syndrome and self-reported gluten intolerance were included as conservative exclusion criteria because they may be associated with elimination diets, dietary restrictions, or altered habitual food choices, rather than because they necessarily impair omega-3 absorption. Participants using omega-3 supplements were excluded to distinguish dietary omega-3 intake from supplemental intake. Those reporting a previous clinical diagnosis of anxiety, depression, or bipolar disorder were excluded to define a non-clinical study sample.

**Figure 1:**
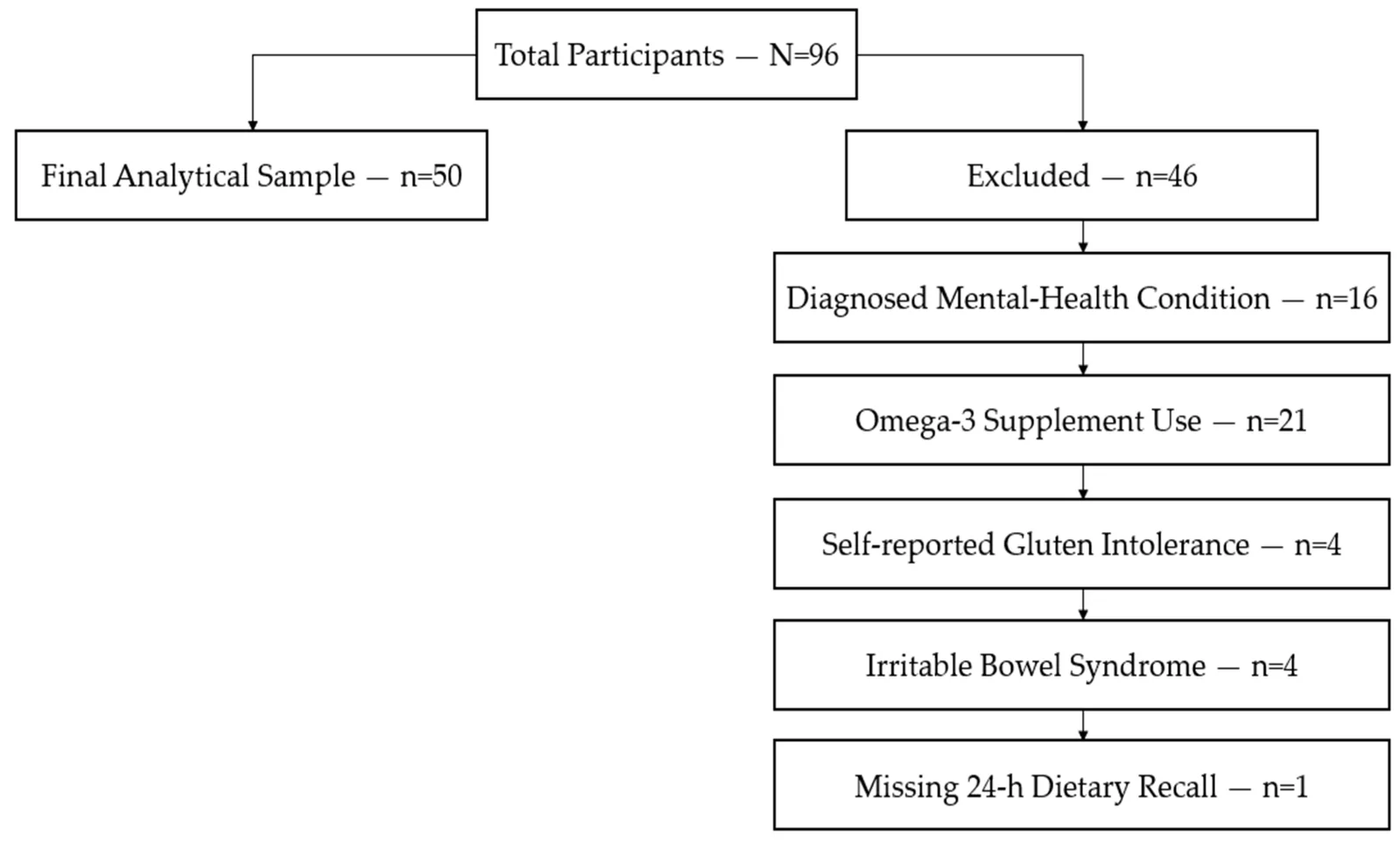
Participant Flowchart. Note: N = total number of participants; n = number of participants in each group.

A total of 96 students were recruited and screened. Eligibility and data-completeness criteria were applied sequentially, and each excluded participant was assigned to the first applicable reason in the screening hierarchy; therefore, the exclusion counts were mutually exclusive. Of the 46 excluded participants, 16 reported a diagnosed mental-health condition, 21 reported omega-3 supplement use, eight reported one of the specified gastrointestinal conditions, and one had a missing 24-h dietary recall. The final analytical sample comprised 50 participants with complete data (52.1% of those recruited). Participant selection is presented in Figure 1.

Because the study was exploratory, recruitment was determined by feasibility, and no formal a priori sample-size calculation was performed. After recruitment and data collection had been completed, a post-recruitment sensitivity calculation was conducted using GPower Version 3.1.9.7 to characterise the magnitude of effects detectable with the final analytical sample [20]. With *n* = 50, a two-sided α level of 0.05, and 80% power, the minimum detectable absolute correlation under the bivariate-normal correlation model was |r| = 0.384. Because GPower does not provide a dedicated procedure for Spearman’s rank correlation, this value was treated as an approximation for the exploratory rank-correlation analyses. For the incremental contribution of estimated dietary EPA + DHA intake in the fully adjusted primary regression model, which included one tested predictor and five predictors in total, the minimum detectable effect was f^2^ = 0.164, corresponding to a partial R^2^ of approximately 0.141. The study therefore had limited sensitivity to detect smaller correlations or independent regression effects. This post-recruitment calculation characterises detectable effect sizes and does not establish that the study was adequately powered for small effects.

### 2.3. Data Collection and Measures

#### 2.3.1. Dietary Assessment

Habitual omega-3 intake was assessed using a food frequency questionnaire adapted from a previously validated omega-3 questionnaire (O3Q) [12,21]. The questionnaire assessed marine sources of EPA and DHA, including fish and seafood, and plant-based sources of ALA, including nuts, seeds, and selected vegetable oils. Participants were also able to report additional omega-3-rich foods not listed in the questionnaire. The complete study-specific instrument and its response options are provided in Supplementary File S1, Appendix A.1.

A single 24-h dietary recall was administered separately from the food-frequency questionnaire to characterise recent dietary intake. Participants were instructed to report all foods and beverages consumed during the preceding 24 h, including the time and place of consumption, food or product details, preparation method, and portion size. The form included a link labelled “View the portion size Guide” to assist participants with portion estimation (Supplementary File S1, Appendix A.2) [22].

#### 2.3.2. Mental Health Assessment

Anxiety symptom severity was assessed using the seven-item Generalized Anxiety Disorder scale (GAD-7), a self-report screening instrument referring to symptoms experienced during the previous two weeks [23]. Each item was rated on a four-point response scale ranging from 0 (“not at all”) to 3 (“nearly every day”), yielding a total score of 0–21. Higher scores indicated greater anxiety symptom severity. Scores were categorized as minimal (0–4), mild (5–9), moderate (10–14), or severe (15–21). The original validation study reported excellent internal consistency (Cronbach’s α = 0.92) [23], while a subsequent validation study conducted in the general population reported a Cronbach’s α of 0.89 [24].

Depressive symptom severity was assessed using the nine-item Patient Health Questionnaire (PHQ-9), which also refers to symptoms experienced during the previous two weeks [25]. Each item was rated from 0 (“not at all”) to 3 (“nearly every day”), yielding a total score of 0–27. Higher scores indicated greater depressive symptom severity. Scores were categorized as minimal (0–4), mild (5–9), moderate (10–14), moderately severe (15–19), or severe (20–27). In the original validation study, the PHQ-9 demonstrated high internal consistency in both primary care (Cronbach’s α = 0.89) and obstetrics–gynecology samples (Cronbach’s α = 0.86) [25]. More recently, a representative general-population study reported a Cronbach’s α of 0.90 (95% CI 0.89–0.91) and a McDonald’s ω of 0.93 (95% CI 0.92–0.94) [26]. In the analytical sample, internal consistency was good for the GAD-7 (Cronbach’s α = 0.872) and acceptable for the PHQ-9 (Cronbach’s α = 0.794).

Both the GAD-7 and PHQ-9 are widely used self-report screening instruments with established reliability and validity in clinical and general-population samples [24,26]. However, scores derived from these instruments indicate symptom severity and do not, by themselves, establish a clinical diagnosis.

#### 2.3.3. Demographic and Lifestyle Variables

The questionnaire collected information on age, sex, academic year, field of study, smoking status, alcohol consumption, physical activity level, height, and body weight. Body Mass Index (BMI) was calculated as weight in kilograms divided by height in metres squared and classified according to World Health Organization categories: underweight, normal weight, overweight, and obesity [27]. Physical activity level was assessed using self-reported weekly activity categories ranging from sedentary to highly active.

### 2.4. Estimation and Reference-Based Categorisation of Omega-3 Intake

Habitual dietary EPA + DHA and ALA intakes used in the statistical analyses were estimated exclusively from the weekly food-frequency component of the adapted O3Q. The 24-h dietary recall was not mathematically combined with the food-frequency estimates and was not used to estimate habitual omega-3 intake. It was used separately to characterise recent dietary intake and to estimate total energy and macronutrient intake. Energy, macronutrient, and fibre intakes from the 24-h dietary recall were calculated using Nutritics dietary analysis software (Research Edition, Nutritics Ltd., Dublin, Ireland) based on the foods, beverages, and portion sizes reported by each participant.

Frequency responses were assigned weekly consumption values as follows: never = 0, less than once per week = 1, 1–2 times per week = 2, 3–4 times per week = 4, and 5 or more times per week = 5 servings/week. For each food item, weekly fatty acid intake was calculated by multiplying the assigned frequency by the fatty acid content of the specified standard portion. Fish and seafood were standardised to 100 g cooked portions; the portions assigned to plant-based foods are reported in Supplementary File S1, Appendix A.1. Weekly EPA and DHA values were summed, converted to milligrams, and divided by seven to obtain estimated dietary EPA + DHA intake in mg/day. Weekly ALA values were divided by seven and expressed as g/day. All FFQ-derived omega-3 calculations were performed in Microsoft Excel.

Food-composition values for individual EPA, DHA, and ALA were obtained from the United States Department of Agriculture FoodData Central database [28]. Food items were matched as closely as possible according to species or food type, preparation method, and cooked or uncooked form. For mixed dishes, only omega-3-containing ingredients for which the food type and portion could be identified were included in the calculation. Vegetable oils were coded according to the reported oil type and amount. Product-specific information was used for fortified or brand-specific foods when sufficiently detailed information was reported, and an appropriate composition value was available; otherwise, the closest generic food entry was used. FoodData Central was selected because it provided preparation-specific, item-level values for individual EPA, DHA, and ALA across the foods included in the adapted questionnaire and enabled the use of a single composition source throughout the calculations. However, no complete recalculation using the UK Composition of Foods Integrated Dataset was performed, and possible differences between the US and UK food supplies are acknowledged as a source of exposure-measurement error.

Reference-based categorisation was used only for exploratory analyses. Estimated dietary EPA + DHA intake was classified using the European Food Safety Authority and Food and Agriculture Organization reference value of 250 mg/day for adults [13,29]. Participants consuming <250 mg/day were classified as below the reference value, whereas those consuming ≥250 mg/day were classified as meeting or exceeding the reference value.

Estimated dietary ALA intake was classified using the sex-specific Adequate Intake values reported by the US National Institutes of Health: 1.6 g/day for men and 1.1 g/day for women [30]. Participants consuming less than the sex-specific value were classified as below the reference value, whereas those meeting or exceeding it were classified as meeting or exceeding the reference value. These categories were used as nutritional reference classifications rather than as diagnostic cut-offs or thresholds for anxiety or depression.

### 2.5. Statistical Analysis

IBM SPSS Statistics Version 29.0.2.0 (IBM Corp., Armonk, NY, USA) and Python 3.12.13, SciPy 1.17.0, NumPy 2.3.5 were used for data management and statistical analyses. The analytical sample comprised 50 participants with complete data. The distributions of continuous variables were assessed using the Shapiro–Wilk test. Continuous variables were summarized using means and standard deviations together with medians and interquartile ranges. Categorical variables were reported as frequencies and percentages.

The association between estimated dietary EPA + DHA intake and GAD-7 total score was designated as the primary exposure–outcome analysis. GAD-7 was analysed as a continuous score ranging from 0 to 21, with higher scores indicating greater anxiety symptom severity. Estimated dietary EPA + DHA intake was divided by 100 so that the regression coefficient represented the estimated difference in GAD-7 score per 100 mg/day higher estimated dietary EPA + DHA intake. Sequential ordinary least-squares regression models were fitted. Model 0 included estimated dietary EPA + DHA intake alone; Model 1 additionally included age and sex; Model 2 additionally included total energy intake; and Model 3 additionally included alcohol use. Model 3 was considered the fully adjusted primary model. Age was entered continuously in years. Total energy intake was divided by 100 so that its coefficient represented the estimated difference associated with a 100 kcal/day higher intake. Sex was coded as male = 0 and female = 1, with male participants as the reference group. Alcohol use was coded as no = 0 and yes = 1, with no alcohol use as the reference group. All specified covariates were retained irrespective of their statistical significance, and no stepwise variable-selection procedure was applied.

HC3 heteroskedasticity-consistent standard errors, 95% confidence intervals, and *p* values were reported for the regression coefficients. Unstandardized coefficients, standardized beta coefficients, R^2^, adjusted R^2^, residual degrees of freedom, and variance inflation factors were also calculated. Regression assumptions and model stability were assessed using the Ramsey RESET test for functional form, the Shapiro–Wilk test for residual normality, the Breusch–Pagan test for heteroscedasticity, and variance inflation factors for multicollinearity. Potentially influential observations were screened using an absolute studentized deleted residual greater than 3, Cook’s distance greater than 4/N, and leverage greater than 2p/N, where p represented the number of model parameters, including the intercept. All validated observations were retained in the primary analysis.

Sensitivity analyses were conducted by excluding the observation with the largest studentized deleted residual, excluding the observation with both high leverage and elevated Cook’s distance, excluding both observations simultaneously, additionally adjusting Model 3 for continuous BMI, and excluding participants with estimated dietary EPA + DHA intake above 1200 mg/day. An additional model used log_2_(EPA + DHA + 1) as a transformed representation of estimated dietary intake, with the coefficient interpreted as the estimated difference in GAD-7 score associated with a doubling of EPA + DHA + 1 mg/day. Regression diagnostics and sensitivity analyses are reported in Supplementary Table S4.

All analyses involving PHQ-9 scores or estimated dietary ALA intake were considered exploratory. Spearman’s rank correlations were calculated among estimated dietary EPA + DHA intake, estimated dietary ALA intake, GAD-7 scores, and PHQ-9 scores, resulting in six pairwise correlations. Bias-corrected and accelerated 95% confidence intervals were obtained using 5000 bootstrap samples.

Four exploratory Mann–Whitney U tests compared GAD-7 and PHQ-9 scores between participants below and those at or above the reference intake thresholds for estimated dietary EPA + DHA and ALA. Estimated dietary EPA + DHA groups were defined as <250 versus ≥250 mg/day. Estimated dietary ALA groups were defined using sex-specific reference values of 1.6 g/day for men and 1.1 g/day for women. Two-sided exact *p* values based on the conditional permutation distribution, with the observed tie structure retained, were reported. Rank–biserial correlations were calculated as effect-size estimates, with 95% confidence intervals based on 5000 stratified bootstrap samples.

For the categorical exploratory analyses, the original GAD-7 and PHQ-9 severity categories were collapsed into minimal or mild symptoms (scores 0–9) and moderate or greater symptoms (scores ≥ 10). Because of sparse expected cell counts, four two-sided Fisher’s exact tests were used to examine the associations between the two reference-based estimated dietary omega-3 intake classifications and the two symptom-severity classifications. Conditional odds ratios and 95% confidence intervals were reported. The Benjamini–Hochberg procedure was applied separately to the six exploratory correlations, four Mann–Whitney comparisons, and four Fisher’s exact tests. False-discovery-rate-adjusted q values were reported alongside the corresponding unadjusted *p* values. All statistical tests were two-sided. Statistical significance for the primary analysis was defined as *p* < 0.05. Exploratory findings were interpreted using effect estimates, confidence intervals, and FDR-adjusted q values rather than isolated unadjusted *p* values.

## 3. Results

### 3.1. Participant Flow and Sample Characteristics

A total of 96 university students were recruited for the study. A total of 50 participants (52.1% of those recruited) were retained for the final analysis after applying the exclusion criteria. Forty-six participants were excluded due to diagnosed mental health conditions (*n* = 16), omega-3 supplementation (*n* = 21), gastrointestinal conditions including gluten intolerance or irritable bowel syndrome (*n* = 8), or missing 24-h dietary recall data (*n* = 1). The inclusion and exclusion criteria for participant selection are shown in Figure 1.

The descriptive characteristics of the analytical sample are presented in Table 1. The mean age of the participants was 25.36 ± 6.68 years, 70.0% were female, 52.0% were master’s students, and 38.0% were studying nutrition. Mean estimated dietary EPA + DHA and ALA intakes were 472.92 ± 366.46 mg/day and 0.96 ± 0.76 g/day, respectively. Mean GAD-7 and PHQ-9 scores were 6.66 ± 4.73 and 7.02 ± 4.76, respectively; 80.0% of participants reported minimal or mild symptoms on each screening measure.

### 3.2. Exploratory Correlations Between Estimated Dietary Omega-3 Fatty Acid Intake and Mental-Health Screening Scores

Exploratory Spearman correlation analyses are presented in Supplementary Table S1. Estimated dietary EPA + DHA intake showed a weak inverse correlation with GAD-7 score (r_s_ = −0.169, BCa 95% CI: −0.476 to 0.159; *p* = 0.240, FDR q = 0.360). No other omega-3–symptom correlation was statistically significant, and all corresponding 95% confidence intervals included zero.

### 3.3. Association Between Estimated Dietary EPA + DHA Intake and GAD-7 Score

Sequential linear regression models with HC3 robust standard errors are presented in Table 2. In the fully adjusted primary model (Model 3), each additional 100 mg/day of estimated dietary EPA + DHA intake was associated with a 0.325-point lower GAD-7 score (B = −0.325, HC3 SE = 0.198, 95% CI: −0.725 to 0.074; standardized β = −0.252); however, the association was not statistically significant (*p* = 0.108). Model 3 explained 19.7% of the observed variance in GAD-7 scores (adjusted R^2^ = 0.106).

The estimated dietary EPA + DHA coefficient was inverse and statistically significant in Model 0 (B = −0.360; *p* = 0.020), Model 1 adjusted for age and sex (B = −0.390; *p* = 0.022), and Model 2 additionally adjusted for total energy intake (B = −0.387; *p* = 0.025), but attenuated after alcohol use was added in Model 3. Female participants had higher GAD-7 scores than male participants in Model 3 (B = 2.921; 95% CI: 0.131 to 5.710; *p* = 0.041), whereas age, energy intake, and alcohol use were not statistically significant covariates.

Model diagnostics and sensitivity analyses are reported in Supplementary Table S4. The estimated dietary EPA + DHA coefficient remained inverse but statistically non-significant after exclusion of potentially influential observations, additional adjustment for BMI, and exclusion of participants with estimated dietary EPA + DHA intake > 1200 mg/day. After log_2_ transformation, the coefficient reversed direction and remained non-significant (B = 0.166; 95% CI: −0.398 to 0.730; *p* = 0.557). The loss of statistical significance after full adjustment and this direction reversal argue against interpreting the inverse linear-model coefficient as evidence of a dose–response relationship.

### 3.4. Exploratory Comparisons Based on Estimated Dietary Omega-3 Intake Thresholds

Exploratory comparisons based on reference intake thresholds are presented in Supplementary Tables S2 and S3. GAD-7 and PHQ-9 scores did not differ significantly between participants below and those meeting or exceeding the estimated dietary EPA + DHA or sex-specific estimated dietary ALA reference values. Similarly, no statistically significant associations were observed between reference-based omega-3 intake classifications and collapsed symptom-severity categories in Fisher’s exact tests. All corresponding effect-size confidence intervals included the null value, and no finding remained significant after FDR adjustment.

## 4. Discussion

In this small exploratory cross-sectional study, higher estimated dietary EPA + DHA intake was associated with lower GAD-7 scores in the fully adjusted primary model, but the estimate was imprecise and not statistically significant (B = −0.325 per 100 mg/day; 95% CI: −0.725 to 0.074; *p* = 0.108). Although the coefficient was inverse and statistically significant in less-adjusted models, it attenuated after alcohol use was added and showed sensitivity to the functional form of the exposure. Exploratory correlations and reference-based comparisons involving estimated dietary EPA + DHA, estimated dietary ALA, GAD-7, and PHQ-9 were also non-significant. Taken together, these preliminary findings do not provide conclusive evidence of an independent association between estimated dietary EPA + DHA intake and anxiety symptoms and should be considered hypothesis-generating.

The direction of the estimated dietary EPA + DHA coefficient in the primary linear model is broadly consistent with the literature suggesting that long-chain omega-3 fatty acids may be relevant to anxiety-related outcomes [8,10,18]. EPA and DHA may influence neuronal membrane function, neurotransmitter signalling, hypothalamic–pituitary–adrenal axis regulation, and inflammatory processes implicated in anxiety and depression [4,5,9]. However, these mechanisms were not measured in the present study and therefore cannot be used to explain the observed coefficient. Furthermore, much of the supportive evidence comes from supplementation trials or clinical populations, whereas the present study assessed estimated dietary intake in a non-clinical student sample. Differences in population characteristics, baseline omega-3 status, EPA-to-DHA composition, intake level, and duration of exposure limit direct comparisons with intervention studies.

The lack of consistency across the analytical approaches is important. The statistically significant inverse associations observed in the less-adjusted linear models were not corroborated by statistically significant rank-based correlations or reference-threshold comparisons. These methods evaluate different features of the data and have different assumptions and statistical efficiency, particularly in a small sample with a skewed exposure distribution and several comparatively high intake values. In addition, the 250 mg/day threshold was developed as a general-health reference value rather than as a threshold for anxiety or depression prevention [13,29]. Dichotomising intake also reduces information and resulted in unequal group sizes. Consequently, the absence of differences across reference-threshold groups should not be interpreted as evidence that the reference value is irrelevant to health; equally, the inverse coefficient from the continuous linear model should not be interpreted as demonstrating a protective dose–response relationship. Although the Ramsey RESET test did not indicate functional-form misspecification in the primary model, the reversal of the coefficient after log_2_ transformation shows that the estimated association was sensitive to how estimated dietary EPA + DHA intake was represented and provides no consistent support for a dose–response interpretation.

No statistically significant associations were identified between estimated dietary EPA + DHA intake and PHQ-9 scores in the exploratory analyses. Previous observational and intervention evidence concerning omega-3 intake and depressive symptoms has also been heterogeneous [6,19,31,32,33]. Differences in study population, baseline symptom severity, dietary versus supplemental exposure, omega-3 formulation, and assessment methods may contribute to variation across studies. The present null findings should nevertheless be interpreted primarily in light of the limited sample size and measurement uncertainty rather than as evidence that EPA and DHA have no relationship with depressive symptoms.

Estimated dietary ALA intake was also not significantly associated with either GAD-7 or PHQ-9 scores, and the corresponding exploratory effect estimates were small with confidence intervals that included zero. ALA differs from EPA and DHA in its dietary sources and metabolism, and its conversion into long-chain omega-3 fatty acids is limited and influenced by the wider dietary fatty acid profile [11,34]. Competition between ALA and linoleic acid for shared metabolic pathways may be relevant, but omega-6 intake was not assessed in this study. Therefore, the ALA findings cannot distinguish between the absence of an association, insufficient statistical sensitivity, exposure-measurement error, or variation in the conversion of ALA into longer-chain metabolites.

Female participants had higher GAD-7 scores than male participants in the fully adjusted model. This direction is consistent with reports of higher anxiety symptom scores among female participants in some medical-student samples [35,36]. However, sex was included as a covariate rather than as a primary exposure, the sample was predominantly female, and the study was not designed or powered to estimate sex differences. This secondary coefficient should therefore be interpreted cautiously and should not be presented as establishing an independent sex effect. Age, total energy intake, and alcohol use were not statistically significantly associated with GAD-7 scores in the fully adjusted model.

The study has several strengths. It examined estimated dietary EPA + DHA and ALA separately rather than combining all omega-3 fatty acids into a single exposure. Anxiety and depressive symptoms were measured using established screening instruments [23,25], and the revised analysis distinguished a single primary exposure–outcome association from exploratory analyses. The sequential modelling strategy retained specified covariates irrespective of statistical significance and avoided data-driven stepwise variable selection. HC3 robust standard errors, confidence intervals, model diagnostics, influence screening, exact tests, bootstrap confidence intervals, false-discovery-rate adjustment, and sensitivity analyses were used to provide a more transparent assessment of uncertainty and model stability.

Several limitations must also be acknowledged. First, the final analytical sample was small, and no formal a priori sample-size calculation was conducted. The post-recruitment sensitivity calculation indicated that, with *n* = 50, approximately 80% power was available only for correlations of |r| ≥ 0.384 under its assumptions; it did not establish adequate power for small effects. Smaller but potentially meaningful associations may therefore have been missed, increasing the risk of type II error and producing imprecise regression estimates. Conversely, the use of multiple model specifications, sensitivity analyses, and exploratory tests may have increased the possibility of chance findings and type I error, although false-discovery-rate correction was applied separately within the exploratory test families. Although the covariate set was deliberately restricted and no stepwise variable-selection procedure was used, overfitting and model instability cannot be excluded given the limited number of observations relative to the fitted parameters. Second, the cross-sectional design precludes causal or temporal inference. Estimated dietary omega-3 intake may influence anxiety symptoms, but anxiety could also affect food selection, appetite, meal regularity, or fish consumption. Residual confounding by socioeconomic circumstances, sleep, habitual caffeine intake, medication use, family psychiatric history, recent stressful life events, adiposity-related metabolic factors, overall dietary pattern, or other health behaviours also remains possible. Obesity and depression have a bidirectional relationship and may share inflammatory, neuroendocrine, adipokine, and lipokine pathways [37], but these mechanisms were not directly assessed in the present study. Third, 46 of the 96 recruited participants were excluded, so only 52.1% of those recruited entered the analysis; recruitment also relied on convenience and snowball sampling. This degree of participant selection increases the potential for selection bias and limits generalisability. Excluding participants with diagnosed mental health conditions, omega-3 supplement use, selected gastrointestinal conditions, or incomplete dietary data produced a selected, non-clinical sample and may have restricted the range of both exposure and symptom scores. Self-selection may also have favoured students with an interest in nutrition or mental health. The sample was predominantly female, and nutrition was the largest field of study. Moreover, 52.0% of the participants were master’s students. Academic stress has been associated with poorer academic achievement among medical postgraduates [38], work-organization factors have been linked to mental-health problems among PhD students [39], and a substantial burden of moderate or severe depression or anxiety symptoms has been reported among economics graduate students [40]. However, academic stress, supervisory and organizational conditions, and stage of postgraduate study were not assessed in the present study and may represent additional sources of unmeasured variation. Information on university attended, ethnicity, socioeconomic background, domestic or international student status, and broader dietary pattern was not collected. The findings should therefore not be considered representative of, or generalised to, the wider UK university-student population. Fourth, dietary intake was self-reported and may have been affected by recall error, portion-size error, social-desirability bias, and within-person variation. The adapted omega-3 questionnaire was based on the O3Q, but it was not independently revalidated after modification, and the changes may have altered its measurement properties. The single 24-h recall used to estimate energy and macronutrient intake may not have represented usual covariate intake. Habitual dietary omega-3 intake was estimated from the adapted FFQ, but frequency and portion-size reporting may still have misclassified episodically consumed foods such as oily fish. Estimated intake may also have been affected by incomplete reporting of mixed dishes, cooking oils, fortified foods, and brand-specific products. In addition, food-composition values were derived from USDA FoodData Central rather than a UK-specific database such as CoFID. Differences in fish species, food products, fortification practices, preparation methods, and portion conventions may have introduced additional exposure misclassification. Finally, GAD-7 and PHQ-9 scores measure recent symptom severity and are screening measures rather than clinical diagnoses [26]. No blood-based omega-3 biomarkers were available to validate dietary estimates, and the study did not assess omega-6 intake, the omega-6-to-omega-3 ratio, inflammatory biomarkers, or overall diet quality. These omissions limit biological interpretation and make it difficult to separate omega-3-specific associations from the influence of the wider dietary pattern.

Future research should use larger and more diverse samples, prospectively specified analytical models, and longitudinal or intervention designs. Repeated dietary assessments, UK-specific food-composition data, validated dietary instruments, and biomarkers such as erythrocyte or plasma omega-3 status would improve exposure assessment. Assessment of omega-6 intake, overall diet quality, sleep, stress, and socioeconomic factors would also allow more comprehensive control of potential confounding.

## 5. Conclusions

In this small exploratory cross-sectional study of UK university students, estimated dietary EPA + DHA intake showed an inverse but statistically non-significant association with GAD-7 scores in the fully adjusted primary model. The inverse coefficient lost statistical significance after full adjustment and reversed direction while remaining non-significant after log transformation. Therefore, these preliminary findings do not provide conclusive evidence of an independent association between estimated dietary EPA + DHA intake and anxiety symptom severity and should not be interpreted as evidence of a dose–response relationship. Exploratory analyses also did not identify statistically significant associations of estimated dietary EPA + DHA or ALA intake with anxiety or depressive symptom measures. These findings should be considered hypothesis-generating given the small, selected analytical sample (50 of 96 recruited participants), cross-sectional design, and uncertainty inherent in self-reported dietary assessment. Future studies should include larger and more diverse samples, longitudinal or intervention designs, repeated and validated dietary assessments, UK-specific food-composition data, and biomarker-based measures of omega-3 status. Assessing omega-6 intake, overall diet quality, inflammatory biomarkers, sleep, stress, and socioeconomic factors would further clarify whether omega-3 fatty acid intake is independently related to mental health among university students.

## Figures and Tables

**Table 1:** Descriptive characteristics of the analytical sample (*n* = 50).

Characteristic	*n* (%) or Mean ± SD	Median [IQR]
*Demographic and lifestyle characteristics*		
Age, years	25.36 ± 6.68	24.00 [21.00–26.25]
Sex		
Female	35 (70.0)	
Male	15 (30.0)	
BMI, kg/m^2^	23.84 ± 5.63	22.91 [20.54–25.04]
Alcohol use, yes	19 (38.0)	
Smoking, yes	7 (14.0)	
Physical activity		
Sedentary	5 (10.0)	
Lightly active	17 (34.0)	
Moderately active	20 (40.0)	
Very active	8 (16.0)	
*Academic characteristics*		
Academic year		
Undergraduate year 1	6 (12.0)	
Undergraduate year 2	4 (8.0)	
Undergraduate year 3	7 (14.0)	
Undergraduate year 4	5 (10.0)	
Master’s	26 (52.0)	
PhD	2 (4.0)	
Field of study		
Nutrition	19 (38.0)	
Computer Science	4 (8.0)	
Engineering	3 (6.0)	
Medicine	3 (6.0)	
Business	2 (4.0)	
Finance	2 (4.0)	
Food Science	2 (4.0)	
Pharmacy	2 (4.0)	
Other fields (one participant each)	13 (26.0)	
*Dietary variables*		
Estimated dietary EPA + DHA intake, mg/day	472.92 ± 366.46	337.00 [249.36–656.13]
Estimated dietary ALA intake, g/day	0.96 ± 0.76	0.75 [0.36–1.45]
Total energy intake, kcal/day	1696.48 ± 676.93	1643.50 [1299.00–2006.00]
Estimated dietary protein intake, g/day	99.80 ± 42.74	92.00 [68.75–126.25]
Estimated dietary carbohydrate intake, g/day	176.84 ± 87.80	168.50 [115.50–219.50]
Estimated dietary fat intake, g/day	65.55 ± 36.49	64.00 [41.75–85.00]
Estimated dietary fibre intake, g/day	16.70 ± 9.34	15.60 [9.62–22.18]
Estimated dietary EPA + DHA intake ≥ 250 mg/day	38 (76.0)	
Estimated dietary ALA intake at or above sex-specific threshold	15 (30.0)	
*Mental-health screening scores*		
GAD-7 score	6.66 ± 4.73	5.50 [3.00–9.00]
Minimal (0–4)	20 (40.0)	
Mild (5–9)	20 (40.0)	
Moderate (10–14)	6 (12.0)	
Severe (15–21)	4 (8.0)	
PHQ-9 score	7.02 ± 4.76	6.00 [4.00–9.00]
Minimal (0–4)	17 (34.0)	
Mild (5–9)	23 (46.0)	
Moderate (10–14)	6 (12.0)	
Moderately severe (15–19)	4 (8.0)	
Severe (20–27)	0 (0.0)	

Data are *n* (%) for categorical variables and mean ± standard deviation (SD) with median [interquartile range (IQR)] for continuous variables. The sex-specific ALA thresholds were 1.6 g/day for men and 1.1 g/day for women. Other single-participant fields were Architecture, Arts, Global Human Resources, Health Psychology, History, Human Resources, Information Technology, Law, Marketing and Communication, Medical Genomics, Project Management, Sports Science, and Technology. GAD-7 = Generalized Anxiety Disorder-7; PHQ-9 = Patient Health Questionnaire-9; BMI = body mass index; EPA = eicosapentaenoic acid; DHA = docosahexaenoic acid; ALA = alpha-linolenic acid.

**Table 2:** Sequential linear regression models with HC3 robust standard errors for the association between estimated dietary EPA + DHA intake and GAD-7 score (*n* = 50). (**A**) Model specification and fit. (**B**) Estimated dietary EPA + DHA coefficient across sequential models. (**C**) Full parameter estimates for Model 3.

(**A**)						
*Model*	*Adjustment Set*		*n*	*Residual df*	*R^2^*	*Adjusted R* ^2^
Model 0	Estimated dietary EPA + DHA intake only		50	48	0.078	0.059
Model 1	Model 0 + age and sex		50	46	0.145	0.089
Model 2	Model 1 + total energy intake		50	45	0.147	0.071
Model 3	Model 2 + alcohol use		50	44	0.197	0.106
**(B)**						
*Model*	*B per 100 mg/day*		*HC3 SE*	*95% CI*	*Standardized Beta*	*p*
Model 0	−0.360		0.150	−0.661 to −0.059	−0.279	0.020
Model 1	−0.390		0.164	−0.720 to −0.059	−0.302	0.022
Model 2	−0.387		0.167	−0.723 to −0.051	−0.300	0.025
Model 3	−0.325		0.198	−0.725 to 0.074	−0.252	0.108
**(C)**						
*Variable*	*B*	*HC3 SE*	*95% CI*	*Standardized Beta*	*VIF*	*p*
Intercept	7.028	2.933	1.116 to 12.940	-	-	0.021
Estimated dietary EPA + DHA intake, per 100 mg/day	−0.325	0.198	−0.725 to 0.074	−0.252	1.167	0.108
Age, years	−0.025	0.067	−0.160 to 0.109	−0.035	1.172	0.709
Female vs. male	2.921	1.384	0.131 to 5.710	0.286	1.092	0.041
Energy intake, per 100 kcal/day	0.035	0.134	−0.235 to 0.306	0.051	1.086	0.794
Alcohol use, yes vs. no	−2.202	1.529	−5.284 to 0.879	−0.228	1.045	0.157

B represents the estimated difference in GAD-7 score associated with a one-unit increase in each variable; for estimated dietary EPA + DHA, one unit corresponds to 100 mg/day. Model 3 was the fully adjusted primary model. HC3 heteroskedasticity-consistent standard errors, confidence intervals, and *p* values are reported. R^2^, adjusted R^2^, standardized beta coefficients, and variance inflation factors (VIFs) were obtained from the corresponding ordinary least-squares models. Male sex and no alcohol use were the reference categories. GAD-7 = Generalized Anxiety Disorder-7; EPA = eicosapentaenoic acid; DHA = docosahexaenoic acid.

## Data Availability

The data presented in this study are available on reasonable request from the corresponding author. The data are not publicly available due to privacy and ethical restrictions.

## Supplementary Materials

The following supporting information can be downloaded at: https://www.mdpi.com/article/10.3390/nu18172858/s1, Table S1: exploratory Spearman correlations; Table S2: reference-based group comparisons; Table S3: collapsed symptom-severity contingency analyses; Table S4: regression diagnostics and sensitivity analyses; Supplementary File S1: STROBE Statement—checklist of items that should be included in reports of observational studies.

## Appendix A

Dietary Assessment Instruments

The dietary components administered to the main study cohort are reproduced below. Wording and response categories have been retained, with minor typographic standardisation for supplementary presentation.

### Appendix A.1. Adapted Omega-3 Questionnaire (O3Q)

This study-specific questionnaire was adapted from the O3Q to assess usual dietary sources of EPA, DHA, and ALA. The adapted version was not independently revalidated.

A. Fish and seafood

How often do you consume the following fish and seafood (per 100 g cooked portion)? Please select the most appropriate option for each item. Choose ‘Never’ if you do not consume a specific item.

**No.**	**Food Item**	**Standard Portion**	**Never**	**Less than Once/Week**	**1–2 Times/Week**	**3–4 Times/Week**	**5 or More Times/Week**
1	Salmon	100 g cooked					
2	Mackerel	100 g cooked					
3	Sardines	100 g cooked					
4	Tuna (fresh or canned)	100 g cooked					
5	Herring	100 g cooked					
6	Trout	100 g cooked					
7	Cod	100 g cooked					
8	Shrimp	100 g cooked					
9	Oysters	100 g cooked					
10	Mussels	100 g cooked					
11	Crab	100 g cooked					
12	Lobster	100 g cooked					
13	Other oily fish (specify below)	100 g cooked					

If you selected ‘Other oily fish,’ please specify the type of fish:

____________________________________________________________.

B. Omega-3-rich plant-based foods

How often do you consume the following omega-3-rich plant-based foods in a typical week? Please select the most appropriate option for each item. Choose ‘Never’ if you do not consume a specific item.

**No.**	**Food Item**	**Standard Portion**	**Never**	**Less than Once/Week**	**1–2 Times/Week**	**3–4 Times/Week**	**5 or More Times/Week**
1	Flaxseeds	Approx. 1 tbsp or 7 g					
2	Chia seeds	Approx. 1 tbsp or 7 g					
3	Walnuts	Approx. 28 g or a small handful					
4	Hemp seeds	Approx. 1 tbsp or 7 g					
5	Canola oil	Approx. 1 tbsp or 15 mL					
6	Other plant-based sources (specify below)	Not specified					

If you selected ‘Other plant-based sources,’ please specify:

____________________________________________________________.

C. Omega-3 supplement screening

Do you currently take any omega-3 supplement? ☐ Yes ☐ No

Researcher-use coding note (not displayed to participants). Frequency responses were converted to weekly values as follows: Never = 0; Less than once per week = 1; 1–2 times per week = 2; 3–4 times per week = 4; and 5 or more times per week = 5 servings/week. For each food, the assigned weekly frequency was multiplied by the fatty-acid content of the stated standard portion. Weekly totals were divided by seven; EPA + DHA was reported in mg/day and ALA in g/day. Habitual EPA + DHA and ALA estimates were derived from this food-frequency component only. No participant reported consumption under either the “Other plant-based sources” or “Other oily fish” category; therefore, these open-ended categories did not contribute to the calculation of estimated dietary ALA or EPA + DHA intake.

### Appendix A.2. Twenty-Four-Hour Dietary Recall Form

Participants completed a single 24-h dietary recall covering all foods and beverages consumed during the preceding 24 h. The form instructed participants: ‘Please describe everything you ate for the past 24 h. Be as detailed as possible.’ They were asked to report the time and place of consumption, food or product details, preparation method, and portion size. The form also displayed the prompt: ‘Need help estimating portion sizes? View the portion size Guide.’

Analysis note. The 24-h recall was used separately to characterise recent dietary intake and estimate total energy and macronutrient intake. It was not mathematically combined with the FFQ-derived habitual dietary EPA + DHA or ALA estimates.

- Breakfast

Example Response for Breakfast: Time: 7:30 AM Place: Home Foods and Drinks: 2 scrambled eggs, 2 pieces whole-grain toast, 1 cup orange juice. Preparation Method: Eggs cooked with 1 teaspoon olive oil, no salt.

Describe everything you ate and drank for breakfast. Include the time, place, food description, preparation method, and portion size.

- Lunch

Example Response for Lunch: Time: 13:30 Place: University Foods and Drinks: One bowl of salad with 150 g grilled chicken and one small banana (~100 g) Preparation Method: Salad dressing composed of 1 teaspoon olive oil, 1 tablespoon of balsamic vinegar, and a pinch of salt

Describe everything you ate and drank for lunch. Include the time, place, food description, preparation method, and portion size.

- Dinner

Example Response for Dinner: Time: 19:00 Place: Home Foods and Drinks: Grilled salmon fillet (~150 g), 1 medium baked potato (~150 g), 1 cup steamed carrots (~150 g) Preparation Method: Salmon grilled with 1/2 tablespoon of olive oil, a pinch of salt, and black pepper. Carrots and potato were seasoned with 1 teaspoon garlic powder and 1 teaspoon of black pepper, no added fat.

Describe everything you ate and drank for dinner. Include the time, place, food description, preparation method, and portion size.

- Snacks

Example Response for Snack: Time: 15:30 Place: University Foods and Drinks: 1 medium-sized apple (~150 g), a handful of raw, unsalted nuts (~30 g) Preparation Method: Nothing added—null

Describe everything you ate and drank for snacks. Include the time, place, food description, preparation method, and portion size.

- Forgotten-food prompt

Did you consume any other snacks, supplements, desserts, or foods not listed above? ☐ Yes ☐ No

If yes, please describe the additional items, including the time, place, food or supplement description, preparation method, and portion size.

Example Time: 18:00 Place: Gym Food/Drink: Protein shake made with 30 g protein powder and 250 mL water. Preparation method: Mixed in a shaker bottle; no additional ingredients.
