# Supplementary material for: Dietary Omega-3 Fatty Acid Intake, Anxiety, and Depression Among University Students in the UK: An Exploratory Cross-Sectional Study
Source: Nutrients. 2026 Sep 1;18(17):2858. doi: 10.3390/nu18172858 (PMC13567499; doi:10.3390/nu18172858)
Supplement: Supplementary file 1 [file nutrients-18-02858-s001.zip › nutrients-4468572-supplementary.pdf]

Supplementary Table S1. Exploratory Spearman correlations among omega-3 intake and mental-health screening scores (N = 50)

Variable 1	Variable 2	Spearman rho	BCa 95% CI	Unadjusted p	FDR q
EPA+DHA intake	ALA intake	0.332	0.063 to 0.551	0.019	0.056
EPA+DHA intake	GAD-7 score	-0.169	-0.476 to 0.159	0.240	0.360
EPA+DHA intake	PHQ-9 score	-0.176	-0.493 to 0.178	0.222	0.360
ALA intake	GAD-7 score	0.133	-0.142 to 0.412	0.357	0.429
ALA intake	PHQ-9 score	0.082	-0.224 to 0.404	0.572	0.572
GAD-7 score	PHQ-9 score	0.702	0.512 to 0.836	<0.001	<0.001

Confidence intervals were obtained from 5,000 bias-corrected and accelerated (BCa) bootstrap samples. FDR q values were calculated using the Benjamini-Hochberg procedure across the six correlations shown. EPA = eicosapentaenoic acid; DHA = docosahexaenoic acid; ALA = alpha-linolenic acid; GAD-7 = Generalized Anxiety Disorder-7; PHQ-9 = Patient Health Questionnaire-9.

Supplementary Table S2. Exploratory comparisons of GAD-7 and PHQ-9 scores across reference-based omega-3 intake classifications

Classification	Outcome	Lower-intake group, n; median [IQR]	Higher-intake group, n; median [IQR]	Mann–Whitney U	Rank-biserial r [95% bootstrap CI]	Exact p	FDR q
EPA+DHA reference threshold (<250 vs ≥250 mg/day)	GAD-7	12; 6.0 [3.0–14.0]	38; 5.0 [3.0–9.0]	216.0	0.053 [−0.397 to 0.485]	0.791	0.969
EPA+DHA reference threshold (<250 vs ≥250 mg/day)	PHQ-9	12; 6.5 [3.2–9.0]	38; 6.0 [4.0–9.0]	226.0	−0.009 [−0.428 to 0.382]	0.969	0.969
Sex-specific ALA reference threshold	GAD-7	35; 5.0 [3.0–9.0]	15; 7.0 [4.0–10.0]	229.0	−0.128 [−0.472 to 0.225]	0.484	0.969
Sex-specific ALA reference threshold	PHQ-9	35; 5.0 [4.0–9.0]	15; 8.0 [3.0–10.0]	231.0	−0.120 [−0.465 to 0.242]	0.511	0.969

The EPA+DHA reference groups were defined as <250 and ≥250 mg/day. Sex-specific ALA reference values were 1.6 g/day for men and 1.1 g/day for women. Positive rank-biserial correlations indicate higher symptom scores in the lower-intake group. Effect-size confidence intervals were calculated using 5,000 stratified bootstrap samples. Mann–Whitney p values are conditional exact p values based on the permutation distribution retaining observed ties. FDR-adjusted q values were calculated using the Benjamini–Hochberg procedure across the four comparisons. IQR, interquartile range; FDR, false discovery rate.

Supplementary Table S3. Collapsed symptom-severity contingency analyses and expected cell counts

Comparison	Lower intake: minimal/mild n (expected)	Lower intake: moderate+ n (expected)	Higher intake: minimal/mild n (expected)	Higher intake: moderate+ n (expected)	Conditional OR [95% CI]	Fisher p	FDR q
EPA+DHA threshold x GAD-7 severity	8 (9.60)	4 (2.40)	32 (30.40)	6 (7.60)	0.38 [0.07 to 2.30]	0.225	0.615

EPA+DHA threshold x PHQ-9 severity	10 (9.60)	2 (2.40)	30 (30.40)	8 (7.60)	1.33 [0.21 to 14.86]	1.000	1.000
ALA threshold x GAD-7 severity	29 (28.00)	6 (7.00)	11 (12.00)	4 (3.00)	1.74 [0.30 to 9.09]	0.462	0.615
ALA threshold x PHQ-9 severity	29 (28.00)	6 (7.00)	11 (12.00)	4 (3.00)	1.74 [0.30 to 9.09]	0.462	0.615

Observed counts are followed by Pearson chi-square expected counts in parentheses. Because all tables contained expected counts below 5, two-sided Fisher's exact tests were used. Symptom categories were collapsed as minimal/mild versus moderate or higher. Odds ratios compare the odds of moderate-or-higher symptoms in the higher-intake group with the lower-intake group. FDR q values were calculated across the four Fisher tests. OR = odds ratio; CI = confidence interval.

Supplementary Table S4. Diagnostics and sensitivity analyses for the primary EPA+DHA-GAD-7 regression

Panel A. Model diagnostics

Assumption/feature	Diagnostic	Statistic	p	Interpretation
Linearity / functional form	Ramsey RESET	F(2, 42) = 0.164	0.849	No evidence of omitted polynomial fitted-value terms
Residual normality	Shapiro-Wilk	W = 0.943	0.018	Evidence of non-normal residuals
Homoscedasticity	Breusch-Pagan	LM(5) = 5.779	0.328	No statistical evidence of heteroscedasticity
Multicollinearity	Maximum VIF	1.172	-	Below conventional concern thresholds
Outlying residual	Maximum absolute studentized deleted residual	3.640	-	Exceeded screening threshold of 3.0
Influence	Maximum Cook's distance	0.275	-	Exceeded 4/N threshold of 0.080
Leverage	Maximum hat value	0.449	-	Exceeded 2p/N threshold of 0.240

Panel B. Sensitivity analyses

Analysis	N	Exposure contrast	B	HC3 SE	95% CI	p	R^2	Adjusted R^2
Primary fully adjusted model	50	Per 100 mg/day	-0.325	0.198	-0.725 to 0.074	0.108	0.197	0.106
Large studentized deleted residual excluded	49	Per 100 mg/day	-0.228	0.179	-0.588 to 0.132	0.209	0.241	0.152
High leverage and Cook's distance observation excluded	49	Per 100 mg/day	-0.340	0.195	-0.733 to 0.053	0.089	0.224	0.134
Both potentially influential observations excluded	48	Per 100 mg/day	-0.243	0.175	-0.597 to 0.112	0.174	0.274	0.188
Additionally adjusted for BMI	50	Per 100 mg/day	-0.315	0.195	-0.709 to 0.078	0.113	0.211	0.101
EPA+DHA intake >1200 mg/day excluded	46	Per 100 mg/day	-0.207	0.337	-0.889 to 0.475	0.544	0.152	0.046
Log2(EPA+DHA+1) exposure	50	Per doubling of EPA+DHA+1	0.166	0.280	-0.398 to 0.730	0.557	0.148	0.052

All sensitivity models were adjusted for age, sex, total energy intake, and alcohol use; the BMI model additionally included BMI. The primary model retained all participants. Influence-screening thresholds were |studentized deleted residual| >3, Cook's distance >4/N, and leverage >2p/N, where p includes the intercept. Four participants with estimated EPA+DHA intake >1200 mg/day were excluded in the high-intake sensitivity analysis. The log2 coefficient represents the estimated GAD-7 difference per doubling of EPA+DHA+1 mg/day. HC3 confidence

intervals and p values are reported. The log-transformed estimate changed direction and remained statistically non-significant, indicating functional-form sensitivity. VIF = variance inflation factor.

Supplementary File S1. STROBE Statement—checklist of items that should be included in reports of observational studies.

	Item No.	Recommendation	Page No.	Relevant text from manuscript
**Title and abstract**	1	(*a*) Indicate the study's design with a commonly used term in the title or the abstract	1-2	The title identifies the report as "An Exploratory Cross-Sectional Study," and the Abstract also describes the study as exploratory and cross-sectional.
		(*b*) Provide in the abstract an informative and balanced summary of what was done and what was found	2	The Abstract summarises the study design, study period, dietary and mental-health assessments, primary adjusted model, sample size, principal effect estimate with 95% CI and p value, exploratory findings, sensitivity analyses, and cautious conclusion.
**Introduction**				
Background/rationale	2	Explain the scientific background and rationale for the investigation being reported	2-3	The Introduction describes the mental-health burden among university students, the biological and clinical rationale for omega-3 fatty acids, inconsistent previous evidence, and the limited evidence on habitual dietary EPA+DHA and ALA intake in non-clinical university students.

Objectives	3	State specific objectives, including any prespecified hypotheses	3	The primary objective was to examine the association between estimated habitual dietary EPA+DHA intake and GAD-7 score. Secondary exploratory objectives involved ALA, PHQ-9, and reference-based intake categories. The prespecified hypothesis was that higher EPA+DHA intake would be associated with lower GAD-7 scores.
**Methods**				
Study design	4	Present key elements of study design early in the paper	4	Section 2.1 states that this was an exploratory cross-sectional study conducted using an online questionnaire assessing dietary intake, mental-health symptoms, and demographic/lifestyle characteristics.
Setting	5	Describe the setting, locations, and relevant dates, including periods of recruitment, exposure, follow-up, and data collection	4	The study was conducted among university students residing in the UK between February and May 2025. Recruitment used social media and QR codes displayed in university locations including libraries and cafeterias.
Participants	6	(*a*) *Cohort study*—Give the eligibility criteria, and the sources and methods of selection of participants. Describe methods of follow-up	4	Section 2.2 gives eligibility criteria (age ≥18 years,

		Case-control study—Give the eligibility criteria, and the sources and methods of case ascertainment and control selection. Give the rationale for the choice of cases and controls<br>*Cross-sectional study*—Give the eligibility criteria, and the sources and methods of selection of participants		current enrolment at a UK university, UK residence, informed consent), exclusion criteria, and participant-selection methods. Recruitment used convenience and snowball sampling through social media and university locations.
		(*b*) *Cohort study*—For matched studies, give matching criteria and number of exposed and unexposed<br>*Case-control study*—For matched studies, give matching criteria and the number of controls per case	N/A	Not applicable. This was a cross-sectional study and no matching was used.
Variables	7	Clearly define all outcomes, exposures, predictors, potential confounders, and effect modifiers. Give diagnostic criteria, if applicable	5-7	Outcomes (GAD-7 and PHQ-9), exposures (estimated EPA+DHA and ALA intake), demographic/lifestyle variables, BMI, and the primary adjustment variables (age, sex, total energy intake, alcohol use) are defined. Coding, score ranges, reference categories, and threshold definitions are provided.
Data sources/ measurement	8*	For each variable of interest, give sources of data and details of methods of assessment (measurement). Describe comparability of assessment methods if there is more than one group	5-6	Section 2.3 describes the adapted O3Q, single 24-hour dietary recall, GAD-7, PHQ-9, and demographic/lifestyle measures. Section 2.4 details frequency-to-intake conversion, standard portions, Nutritics analysis,

				USDA FoodData Central matching, and handling of mixed dishes, oils, fortified foods, and brand-specific products.
Bias	9	Describe any efforts to address potential sources of bias	4, 6-8, 15-16	Potential bias was addressed by clearly describing non-probability recruitment and exclusions, standardising dietary calculations and food matching, retaining all specified covariates, using HC3 robust standard errors, influence diagnostics, exact/bootstrapped procedures, FDR correction, and sensitivity analyses. Remaining selection, measurement, and confounding biases are discussed as limitations.
Study size	10	Explain how the study size was arrived at	4-5	Recruitment was feasibility-based and no formal a priori sample-size calculation was performed. A G*Power sensitivity calculation for N=50 is reported, including a minimum detectable absolute correlation of $\|r\|$=0.384 and minimum detectable incremental regression effect f^2=0.164.

Continued on next page

Quantitative variables	11	Explain how quantitative variables were handled in the analyses. If applicable, describe which groupings were chosen and why	6-8	Continuous variables were summarised using means/SDs and medians/IQRs. EPA+DHA was scaled per 100 mg/day and energy per 100 kcal/day in regression. Reference categories were <250 vs ≥250 mg/day for EPA+DHA and sex-specific ALA values (1.6 g/day men; 1.1 g/day women). Symptom categories were collapsed to 0-9 vs ≥10 for categorical exploratory analyses.
Statistical methods	12	(*a*) Describe all statistical methods, including those used to control for confounding	7-8	Section 2.5 describes sequential OLS regression with HC3 robust inference and specified covariates, model diagnostics and influence screening, Spearman correlations with BCa bootstrap CIs, exact Mann-Whitney tests with rank-biserial effect sizes, Fisher's exact tests, and Benjamini-Hochberg FDR correction.
		(*b*) Describe any methods used to examine subgroups and interactions	8, 17-18	No subgroup or interaction regression modelling was performed. Exploratory comparisons were conducted across reference-based EPA+DHA and sex-specific ALA intake groups; these are described in Section 2.5 and reported in Supplementary Table S2.

	(*c*) Explain how missing data were addressed	4, 7-8	The analytical sample comprised 50 participants with complete data. One participant was excluded because the 24-hour dietary recall was missing; no post hoc energy-plausibility exclusion was introduced.
	(*d*) *Cohort study*—If applicable, explain how loss to follow-up was addressed<br>*Case-control study*—If applicable, explain how matching of cases and controls was addressed<br>*Cross-sectional study*—If applicable, describe analytical methods taking account of sampling strategy	4	No probability-based sampling weights or complex-survey adjustments were applicable. The manuscript explicitly states that convenience/snowball recruitment did not use a population-based sampling frame and that the sample should not be considered representative of UK university students.
	(*e*) Describe any sensitivity analyses	7, 19	Sensitivity analyses excluded the largest studentized deleted residual, the high-leverage/elevated-Cook's-distance observation, both observations together, participants with EPA+DHA >1200 mg/day, added BMI to Model 3, and used log2(EPA+DHA+1) as the exposure. Full results are in Supplementary Table S4.
**Results**			

Participants	13*	(a) Report numbers of individuals at each stage of study—eg numbers potentially eligible, examined for eligibility, confirmed eligible, included in the study, completing follow-up, and analysed	4, 8-9	Ninety-six students were recruited and screened; 46 were excluded and 50 were retained in the final analytical sample. The screening hierarchy and mutually exclusive exclusion process are described in Section 2.2 and the participant flow is reported in Results.
		(b) Give reasons for non-participation at each stage	4, 8-9	Reasons for exclusion were diagnosed mental-health condition (n=16), omega-3 supplement use (n=21), gastrointestinal conditions (n=8: four self-reported gluten intolerance and four IBS), and missing 24-hour dietary recall (n=1).
		(c) Consider use of a flow diagram	9	Figure 1 presents the participant flowchart from 96 recruited participants to the final analytical sample of 50, with exclusion categories and counts.
Descriptive data	14*	(a) Give characteristics of study participants (eg demographic, clinical, social) and information on exposures and potential confounders	9-11	Table 1 reports demographic and lifestyle characteristics, academic year and field of study, dietary variables, omega-3 intake estimates and reference classifications, and GAD-7/PHQ-9 scores and symptom categories. Continuous variables are shown as mean ± SD and median [IQR].

		(b) Indicate number of participants with missing data for each variable of interest	4, 8-11	One participant with a missing 24-hour recall was excluded. The final analytical sample comprised 50 participants with complete data, and Table 1 reports N=50 for the analysed variables.
		(c) *Cohort study*—Summarise follow-up time (eg, average and total amount)	N/A	Not applicable. This was a cross-sectional study with no follow-up period.
Outcome data	15*	*Cohort study*—Report numbers of outcome events or summary measures over time	N/A	Not applicable. This was not a cohort study.
		Case-control study—Report numbers in each exposure category, or summary measures of exposure	N/A	Not applicable. This was not a case-control study.
		Cross-sectional study—Report numbers of outcome events or summary measures	9-11	For the cross-sectional outcomes, Table 1 reports GAD-7 and PHQ-9 summary scores and category counts/percentages. GAD-7: 20 minimal, 20 mild, 6 moderate, 4 severe. PHQ-9: 17 minimal, 23 mild, 6 moderate, 4 moderately severe, 0 severe.
Main results	16	(*a*) Give unadjusted estimates and, if applicable, confounder-adjusted estimates and their precision (eg, 95% confidence interval). Make clear which confounders were adjusted for and why they were included	11-13	Section 3.3 and Table 2 report the unadjusted and sequentially adjusted EPA+DHA coefficients with HC3 SEs, 95% CIs, standardized betas, p values, N, residual df, R^2/adjusted R^2, and VIFs. Model 3 adjusts for age, sex, total energy intake, and alcohol use.
		(*b*) Report category boundaries when continuous variables were categorized	6, 8, 13, 17-18	Category boundaries are stated for EPA+DHA (<250 vs ≥250 mg/day), sex-specific ALA

			reference values (1.6 g/day men; 1.1 g/day women), and the collapsed symptom categories (0-9 vs ≥10). These are also shown in Supplementary Tables S2-S3.
	(*c*) If relevant, consider translating estimates of relative risk into absolute risk for a meaningful time period	N/A	Not applicable. Relative-risk estimates over a time period were not calculated in this cross-sectional study.

Continued on next page

Other analyses	17	Report other analyses done—eg analyses of subgroups and interactions, and sensitivity analyses	11-13, 17-19	Other analyses include exploratory Spearman correlations (Supplementary Table S1), reference-threshold Mann-Whitney comparisons (S2), Fisher's exact tests of collapsed symptom categories (S3), and regression diagnostic/sensitivity analyses (S4).
**Discussion**				
Key results	18	Summarise key results with reference to study objectives	13-15	The Discussion summarises the primary finding as an inverse but statistically non-significant and model-dependent EPA+DHA-GAD-7 association, notes null exploratory correlations and threshold comparisons, and relates these findings directly to the study objectives.
Limitations	19	Discuss limitations of the study, taking into account sources of potential bias or imprecision. Discuss both direction and magnitude of any potential bias	15-16	Limitations include small sample size and limited power, type I/type II error, possible overfitting/model instability, cross-sectional design and reverse causation, residual confounding, selected convenience sample, self-reported dietary measurement error, USDA rather than UK-specific composition data, lack of biomarkers, and unmeasured omega-6/diet quality.

Interpretation	20	Give a cautious overall interpretation of results considering objectives, limitations, multiplicity of analyses, results from similar studies, and other relevant evidence	13-16	The Discussion provides a cautious interpretation that integrates the primary and exploratory results, model sensitivity, multiplicity, exposure measurement uncertainty, relevant omega-3 literature, and alternative explanations. The findings are described as hypothesis-generating rather than causal or confirmatory.
Generalisability	21	Discuss the generalisability (external validity) of the study results	15-16	The manuscript states that the predominantly female, nutrition-enriched, convenience sample and extensive exclusions limit external validity. It explicitly states that the findings should not be considered representative of, or generalised to, the wider UK university-student population.
**Other information**				
Funding	22	Give the source of funding and the role of the funders for the present study and, if applicable, for the original study on which the present article is based	20	Funding statement: "The authors received no financial support for the research, authorship, and/or publication of this article." No funder role therefore applied.

*Give information separately for cases and controls in case-control studies and, if applicable, for exposed and unexposed groups in cohort and cross-sectional studies.

Note: An Explanation and Elaboration article discusses each checklist item and gives methodological background and published examples of transparent reporting. The STROBE checklist is best used in conjunction with this article (freely available on the Web sites of PLoS Medicine at http://www.plosmedicine.org/, Annals of Internal Medicine at http://www.annals.org/, and Epidemiology at http://www.epidem.com/). Information on the STROBE Initiative is available at www.strobe-statement.org.
